# Silver Nanoparticles Synthesized Coating with *Zataria Multiflora* Leaves Extract Induced Apoptosis in HeLa Cells Through p53 Activation

**Published:** 2018

**Authors:** Javad Baharara, Tayebe Ramezani, Nasrein Hosseini, Marzieh Mousavi

**Affiliations:** a *Research Center for Animal Development Applied Biology & Biology Department, Mashhad Branch, Islamic Azad University, Mashhad, Iran. *; b *Biology Department, Faculty of sciences, Kharazmi University, Tehran, Iran.*

**Keywords:** Zataria multiflora, Silver nanoparticle, Apoptosis, VEGF-A, MMP-9

## Abstract

The biosynthesis of nanoparticles is widely considered today. This investigation was aimed at the biosynthesis and coating of Ag.NPs with *Zataria multiflora* (*Zm-*Ag.NPs) leaf extract and assessment of its apoptosis promoting effects. The *Zm*-Ag.NPs was characterized by UV-visible and FTIR spectroscopy, TEM, EDS, DLS, and measurement of zeta-potential. Apoptosis induction effects of *Zm-*Ag.NPs were assessed using acridine orange – propidium iodide** (**AO/PI), DAPI staining, caspase3/9 activation assay, and annexinV/PI assay. Changes in P53, matrix metalloproteinases 2 (MMPs), and vascular endothelial growth factor A (VEGF-A) genes expression were also assessed with semi-quantitative RT-PCR. The UV-visible spectroscopy results showed that the surface plasmon resonance band (SRP) for *Zm*-Ag.NPs was about 440 nm, also, FTIR spectroscopy indicated that plant material embedded around *Zm*-Ag.NPs. The TEM images of the samples revealed that the Ag.NPs varied in morphology and also, the presence of silver element was monitored with EDS. The mean size of *Zm*-Ag.NPs was 30 nm. The *Zm*-Ag.NPs reduced cell viability in a dose and time dependent manner (IC_50_ = 15 μg/mL). AO/PI and DAPI staining indicated chromatin fragmentation and annexinV externalization assay using flow cytometer, confirmed promotion of programmed cell death in the treated cells. Apoptosis was induced through caspase 3/9 activation pathway. This promotion of apoptosis effects is not related with P53 gene up regulation. Finally, it was found that *Zm*-Ag.NPs inhibited cancer cell metastasis through a decrease in MMP and VEGFA expression. *Zm*-Ag.NPs acts as carrier of the plant material compound, and can be applied as anticancer agents.

## Introduction

In the developing world, cancer is still problematic and it is difficult to describe the right way to treat it. According to the WHO reports, cancer causes about 7.9 million deaths worldwide each year (1). In addition, possibility of establishing resistance to treatment strategy and tumor growth is enhanced (2). Apoptosis deregulation takes place in various kinds of cancer cell, which makes it difficult to kill tumors, hence agents that activate apoptotic pathways have the potential of effectively treating cancers (3). Apoptosis targets that are being explored for cancer drug discovery include the caspase-dependent apoptosis and this pathway is the best-known in respect of programmed cell death (4). Hence, continuing the search for novel cancer chemotherapeutic agents is necessary. Natural products are important sources of anti-cancer molecules and many successful anti-cancer drugs are natural products or their analogues (5).

 Nanotechnology is a very interesting field, which is related to the credible reports of the fabrication and use of materials with structural character between those of bulk materials and atoms with at least one dimension of nano-size. The fabrication of metal nanoparticles is an expanding field due to their ability to be used in various fields (6). 

Among them is Ag.NPs, which represent unusual biological and physicochemical activities, and, therefore, have been used in the health care industry (7). The special anti-microbial properties of silver nanoparticles have led to the manufacture of various products based on Ag.NPs, such as nano-silver coated wound dressings, implants, and surgical instruments (8). In recent years, anticancer effects of Ag.NPs were taken into consideration (9).

Plants and microbes are applied in the fabrication of nanoparticles. Use of plant extracts for the synthesis of nanoparticles has advantages, such as being cost-effective, eco-friendly, and also taking place of the process in one setup; also, nanoparticles act as carrier in the transfer of materials into cells (10). Medicinal plants have therapeutic properties due to the presence of various complex chemical substances of different compositions, which are found as plant metabolites in certain parts of the plants.

*Zataria multiflora, a* member of the *Lamiacea* family is a well-known folk medicine (11). The main constituents of this plant are phenolic compounds such as carvacrol, thymol and eugenol (12). Recent findings showed a variety of phytochemicals, including phenolics, in these anticancer properties. Both monophenolic and polyphenolic compounds from a large variety of plant foods, spices and beverages have been shown to inhibit or attenuate the initiation, progression, and spread of cancers in cells *in-vitro* and in animals *in-vivo* (2).

In this study, green Ag.NPs was synthesized using *Z. multiflora* leaf extract and the Ag.NPs was used to induce apoptosis in model system cervical carcinoma cells HeLa.

## Experimental

*Materials*

The *Z. multiflora *was collected from local natural sources, and the solutions used were prepared with distilled water and other chemicals of analytical grade.

MTT [3-(4, 5-dimethylthiazol-2-yl) -2, 5-diphenyltetrazolium bromide], Acridine orange, propidium iodide, 4ʹ, 6-diamidino-2-phenylindole (DAPI), and silver nitrate were obtained from Sigma-Aldrich from England. Fetal bovine serum (FBS) and RPMI-1640 culture medium were purchased from Invitrogen, England. The high pure RNA isolation kit was purchased from Roche, Germany, and cDNA synthsised kit from fermentase. For PCR reaction, PARS TUS kit (Iran) and primer from Bioneer (Korea) were obtained. All the solutions were prepared with double distilled water and other chemicals were of analytical grade.

*Extraction Preparation*

*Zataria multiflora *leaves were collected from Mashhad (Khorasan-Razavi, Iran) and identified by the Herbarium Division of the Mashhad University; they were given the voucher specimen number 34516. The leaves were then washed, dried and powdered before being used for extraction. Following this step, 5 g of the leaf powder was added to 100 mL of sterile distilled water and boiled for five minutes. The extract was then filtered through a Whatman filter (paper No.1) and stored at 4 °C for further use.

*The green synthesis of silver nanoparticles*

*Zm*-Ag.NPs was synthesized by reducing 10 mL of silver nitrate solution (1-5 mM) with 100 to 1000 µL of the *Z. multiflora* leaf extract. Change in the color of the solution from pale brown to dark brown indicated the formation of nanoparticles. This process was carried out at a temperature of 40 °C and at pH 7.0. In order to eliminate any free biomass residue or unbound extract from the surfaces of the nanoparticles, the *Zm-*Ag.NPs was centrifuged and repeatedly washed with distilled water. Subsequently, the product was centrifuged at 9000 rpm for 30 min and dried at 45 °C.

*Characterization methods*

The UV-visible absorption spectra of the samples were measured in different concentrations of the plant extract and silver nitrate and at different time intervals using a spectrophotometer (Biotek Epoch, US). For analysis of UV-visible spectra, 100 µL of the sample was put in a 96-well plate and read. The size of the nanoparticles was determined using DLS/zeta potential analysis (Cordovan, Vaso particle, France). 

The best sample base of the UV-visible results was selected for the DLS study. DLS was applied to assess the size of *Zm-*Ag.NPs at 25 °C using 0.894 cp for the viscosity of the medium, and a fixed angle = 90 for the avalanche photo diode (APD) detector and a wavelength of 657 nm for the 50 mW laser zeta-potential of *Zm-*Ag.NPs in water, were evaluated using CAD (Zeta compact, France) zeta sizer. The TEM results (CM-120, Philips) were obtained by depositing a drop of the best sample, based on the DLS results, on a copper mesh coated with a carbon film; the solvent was then evaporated. The size dispensation was obtained by image analysis using the Image J software package 24, counting at least 200 particles for significant and relevant statistics. The FTIR for the leaf of the plant extract and *Zm-*Ag.NPs was obtained in the range of 4000 to 400 cm.^−1^ with a Perkin Elmer spectrophotometer paragon 1000. The dried *Zm-*Ag.NPs sample was placed on a coated carbon film and examined using EDS analysis (In Ca, UK).

*Cell culture assay*

*MTT assay*

About 5×10^4^ HeLa cells were seeded in a 96 well-plate. After 24 h the cells were treated with 5-60 µg/mL *Zm*-Ag.NPs for 24 and 48 h, at which point, the culture media was aspirated off, and 20 µL of the MTT solution (5mg/mL) was added, before being incubated for 4 h in a cell incubator (37 °C, 5% CO_2_). After this, the MTT solution was aspirated off from the wells. 

The formazan (MTT metabolic product) was resuspended in 200 µL isopropanol, and a 96-well plate reader was used to read the absorbance at 570 nm. The cell viability was calculated using the following equation:

$\mathrm{Cell viability}\left(\%\right)=\frac{\mathrm{Absorbance of sampel}}{\mathrm{Absorbance of Control}}\times 100$

Where A treated and A control are the absorbance of the treated and untreated cells, respectively (13) 

*DAPI staining*

The cells were cultured by adding approximately 5×10^3^ on gelatin-coated coverslips placed on tissue culture previously. The cells were treated with 15 µg/mL *Zm*-Ag.NPs. After 48 h 200 µL of 4% paraformaldehyde was added to the fixed cells and incubated for 20 min at room temperature. The wells were washed twice with PBS, stained with 100 ng/mL DAPI for 510 min and finally, washed with PBS, then observed using fluorescence microscopy with DAPI.

*Acridin orange – propidium iodide (AO/PI) staining*

The seeded HeLa cells were treated with 10 and 20 µg/mL for 48 h. For the AO/PI staining, the cells were harvested and re-suspended before 10 µL of the treated cell sample, 10 µL of the AO staining solution and 10 µL of PI staining solution were combined. Then, 10 µL of the stained sample was added to a Cellometer Counting Chamber and analyzed using fluorescence microscopy (Biomed, Korea). 

*Caspase activation assay*

Caspase-3 and 9 activities were assessed using the colorimetric protease assay Abcam Kit following the protocol of the manufacturer. Briefly, the cells were treated with Zm-Ag.NPs. Apoptosis was induced in the cells treated with *Zm-*Ag.NPs for 24 h. After this, the 1-5 × 10^6^ cells were pelleted and re-suspended in 50 μL of chilled Cell Lysis Buffer, before centrifuging for 1 min. The protein concentration was assayed using the Biuret method. For each assay, 100 μg proteins were diluted with 50 μL Cell Lysis Buffer. Finally, the DEVD-p-NA substrate was added and the samples were read at 400 or 405 nm using a microtiter plate reader (Epoch, US). The fold-increase in Caspase 3 activity was determined by comparing with the control groups.

*Annexin: Determination of apoptotic and necrotic cells*

The amount of apoptotic specific hallmark externalization of PS on the HeLa cell surface was assessed using the Annexin-V-FITC staining kit (Abcam), Mannheim, Germany, according to the manufacturer’s instructions. The cells were tested with 10 and 20 µg/mL *Zm-*Ag.NPs for 48 h before harvesting and centrifuging at 200 xg for 5 min. Then, 5 µL Annexin- V-FITC labeling and 5 µL PI solution were added, before incubating for 5 min at 25 °C and analyzing using a flow cytometer (Bd, UK). 

*Gene expression analysis*

Changes in the expression of VEGF, MMP, and P-53 genes were analyzed using RT-PCR. Briefly, the total RNA of the treated MCF-7 cells was isolated using the High Pure RNA Isolation kit according to the manufacturer’s protocol (Roche, Germany). Then, the cDNA was synthesized using the ParsTous Kit, incubated at 65 °C for 5 min followed by the addition of the RT premix. 

The temperature of the synthesis was according to the protocol: incubation at 65 °C for 5 min followed by the addition of the RT premix, then incubation at 25 °C for 10 min, 50 °C for 60 min and 70 °C for 10 min. Finally, 2 µL of the cDNA produced was added to the 10xbuffer, MgCl_2_ 25 mM, dNTP, Taq DNA polymerase and the appropriate forward and reverse primers. Ultimately, RT-PCR was performed: 1 cycle at 95 °C/4 min, 35 cycles at 94 °C/30 s for denaturation, 57 °C/30 s for annealing, 72 °C/30 s for extension, and 1 cycle of 5 min at 72 °C. The primers were used as follows:

The PCR products were observed by electrophoresis in a 2% agarose gel, read using a UV-detector and semi-quantitatively measured from the UV optical density of bands using ImageJ software.

*Statistical analysis*

Statistical assessment of the data was performed using ANOVA one-way analysis. The Tukey test was applied for comparisons as a posttest with the help of SPSS software, and the results are shown as mean ± SD, and *p* < 0.05 was calculated as the minimum level of significance.

## Results and Discussion

*Green synthesis of Zm-Ag.NPs and characterization*

**Figure 1:**
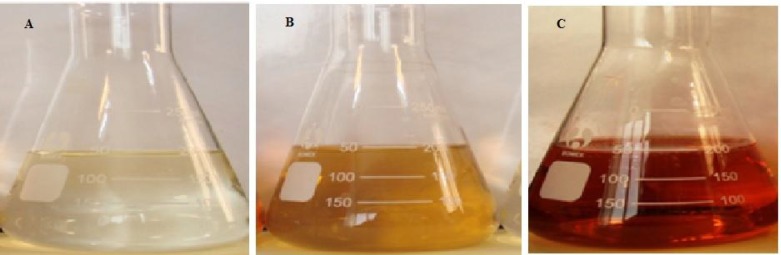
Visual appearance of AgNO_3_ (A), *Zm* extract (B) and *Zm-*Ag.NPs (C

It is well known that *Zm-*Ag.NPs exhibit a brown color in aqua medium. This color appears due to the excitation of the surface plasmon vibrations in the metal nanoparticles. The formation of *Zm-*Ag.NPs was only observed in the mixture containing the AgNO_3_ solution and in the leaf extracts at a maximum absorbance of 440 nm after 4 h. The color change was distinguished by visual observation in the extract of the *Zm* leaf when incubated with AgNO_3_ solution. The color intensity increased with the increased dose of the plant extract at the same duration, at 40 ºC. The color intensity was greatest when 10 mL of AgNO_3_ solution at 2.5 mM and 0.5 mL of the *Zm* leaf extract (10:0.5 v/v) were mixed together. Figure 1A shows the result of the AgNO_3_ solution without the leaf extract, while Figure 1B shows the result of the leaf extract without AgNO_3_, which did not show any significant change in color, and Figure 1C shows *Zm-*Ag.NPs.

*UV-vis analysis of silver nanoparticles*

The UV spectra from the reaction of the reduction of silver ions showed that the NPs have a maximum peak in the absorbance band at the 440 nm wavelength (Figure 2). The fabrication of the nanoparticles was dependent on the different chemical and physical factors, such as the concentration of the metal ions, incubation time and amount of plant extract. In this study, the factors that may affect the incubation time for the synthesis of the *Zm-*Ag.NPs and concentration of silver nitrate were noted. The concentration of silver nitrate is the first factor. When the concentration of silver nitrate increased from 1 to 2.5 Mm, the *Zm-*Ag.NPs absorbance value increased (Figure 1). Synthesis of the silver nanoparticles decreased in the 5 Mm silver nitrate concentration as compared to the 2.5 Mm solution (Figure 2A). Similar results were obtained using the *Anthoceros curcas* extract with different concentrations of silver nitrate (14). 

Figure 2B shows the time-dependent synthesis of *Zm-*Ag.NPs. As the time increased from 1 to 4 h the nanoparticle fabrication also increased. Nanoparticle production was initiated within 40 min at 40 °C. The completion of the nanoparticle synthesis fabrication occurred after 4 h. Other studies also reported that the formation of particles could be affected by altering factors, such as substrate concentration, temperature and time of exposure to the substrate (15). Overall, the best synthesized nanoparticles, 10 mL of AgNO_3 _solution at 2.5 mM and 0.5 mL of *Zm *leaf extract (10:0.5 v/v), were used for future characterizations.

**Table 1:** Primer sequence

**Gene**	**Forward**	**Reverse**
Beta Actin	5CCCGCCGCCAGCTCACCATGG ꞌ3ꞌ	5ꞌAAGGTCTCAAACATGATCTGGGTC3ꞌ
VEGF-A	5ꞌ CCTGCCTTGCTGCTCTACC 3ꞌ	5ꞌ CACACAGGATGGCTTGAAG 3ꞌ
MMP	5ꞌCTGCATCCTCAGCAGGTTG 3ꞌ	5ꞌ GTCTCGGATAGTCTTTATCC3ꞌ
P53	5ꞌ TTGCCGTCCCAAGCAATGGATGA3ꞌ	5ꞌ TCTGGGAAGGGACAGAAGATGAC3ꞌ

**Figure 2A.**
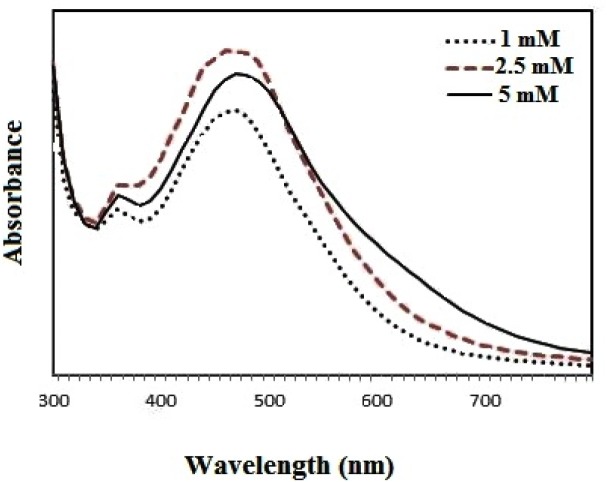
UV-vis absorbance for samples with different concentrations of Ag ions

**Figure 2B:**
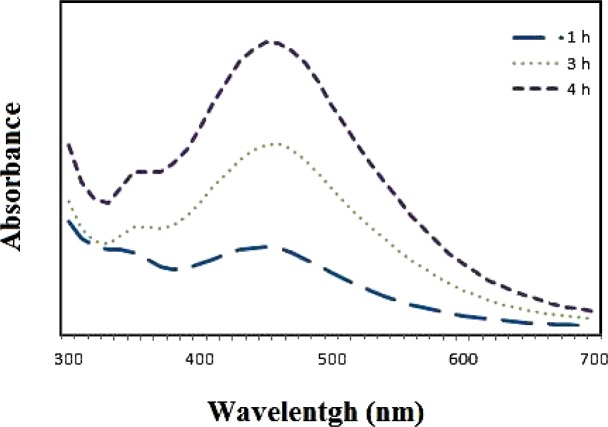
UV-vis absorbance for samples after different incubation times

**Figure 3:**
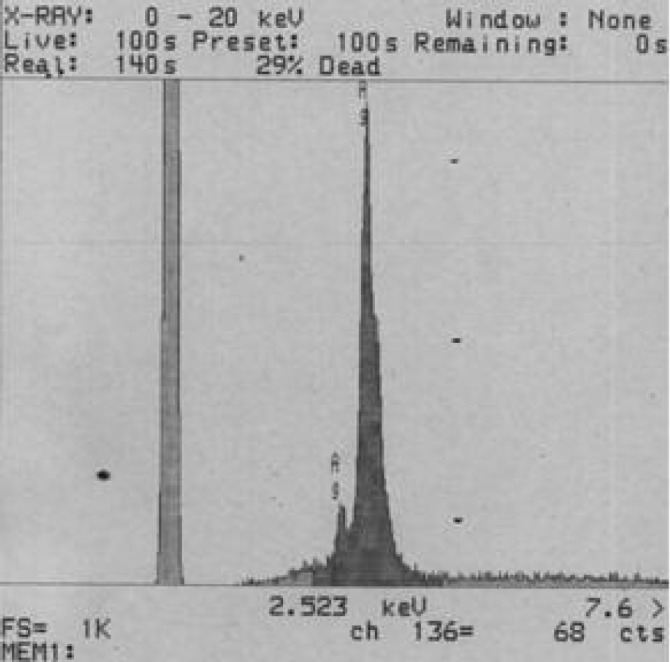
EDS spectrum of bio-synthesized silver nanoparticles

**Figure 4:**
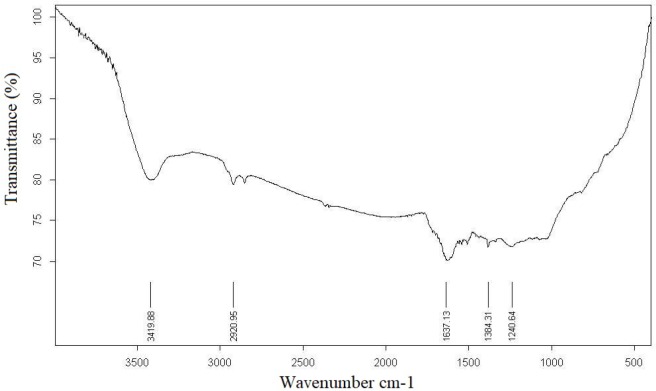
FTIR spectrum of bio-synthesized *Zm*-Ag.NPs

**Figure 5:**
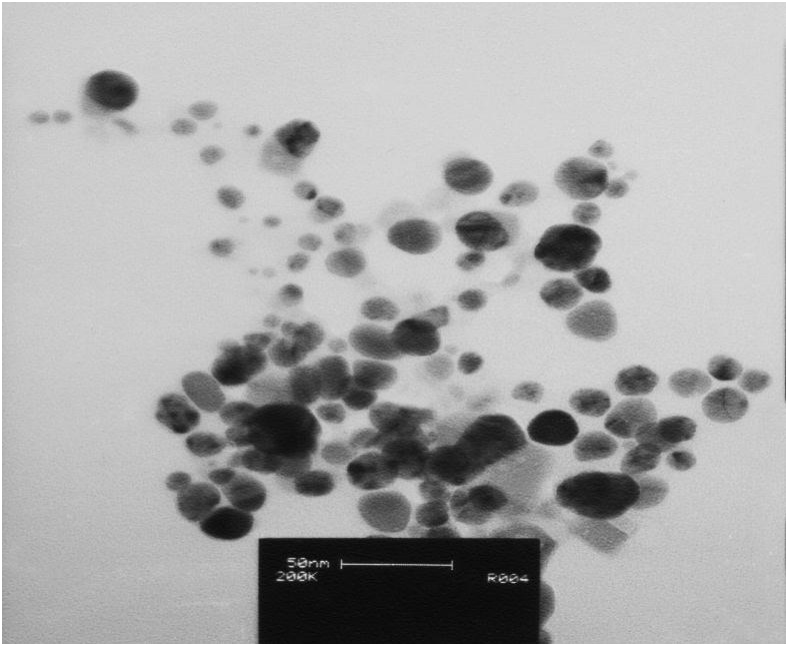
TEM image of the bio-synthesized *Zm*-Ag.NPs

**Figure 6.**
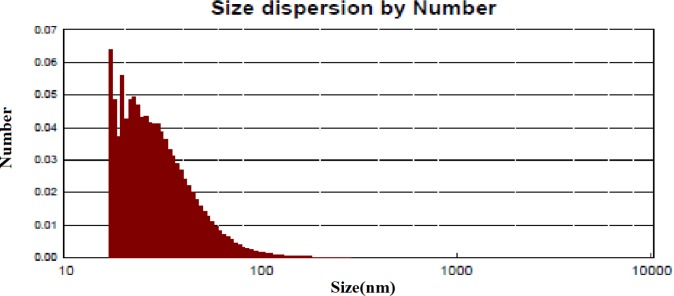
Particle size distribution of bio-synthesized *Zm*-Ag.NPs

**Figure 7:**
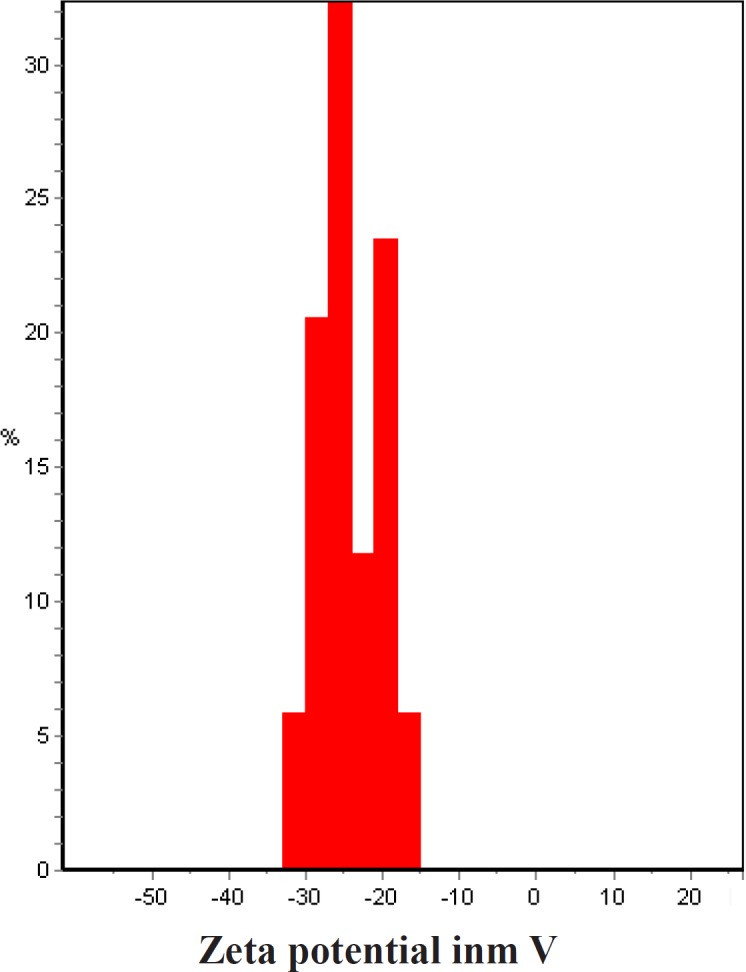
Zeta potential of bio-synthesized *Zm-*Ag.NPs

**Figure 8:**
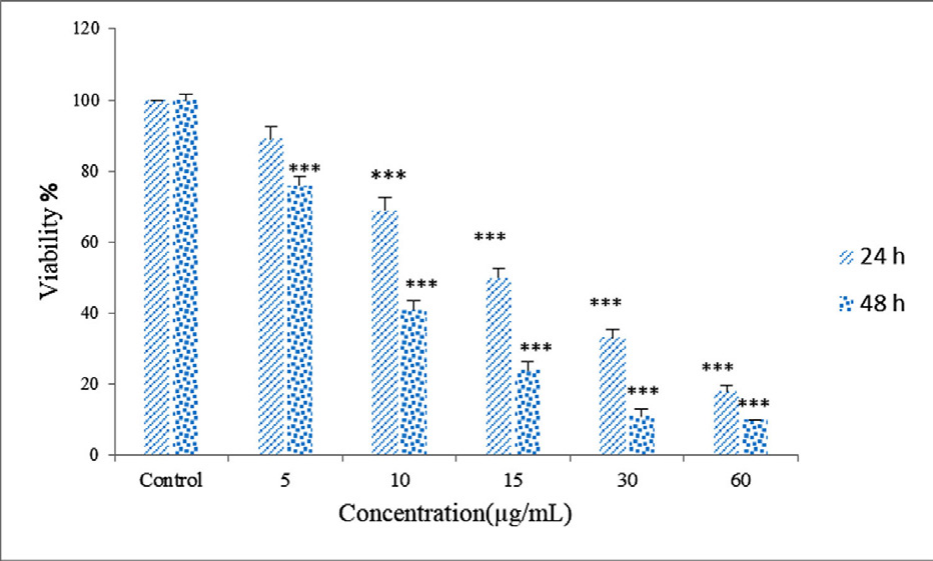
MTT assay,*** p < 0.001

**Figure 9:**
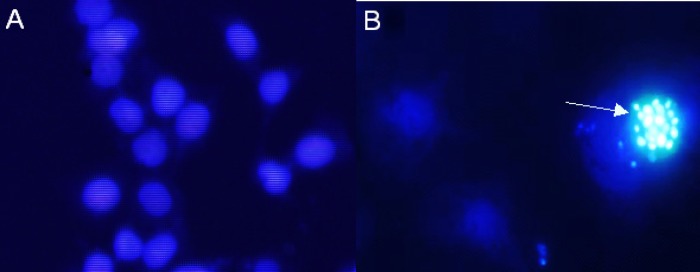
DAPI staining showing total nuclei. Cells were treated with 15 μg/mL Zm-Ag.NPs for 48 h. Apoptotic body formation of breakdown in chromatin (arrow) as a hallmark indicator of apoptosis was observed by DAPI staining under fluorescence microscopy

**Figure 10:**
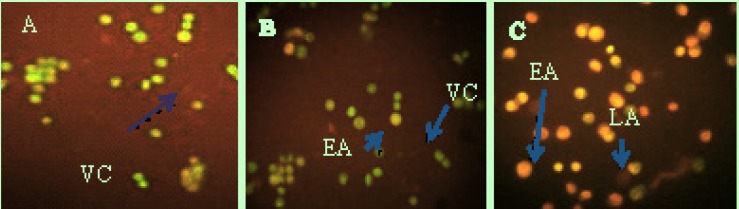
Fluorescent micrograph of A0/PI double-stained HeLa cells. (A) Control group show normal structure. (b) Early apoptosis features were observed after 48 h, representing intercalated acridine orange (green) amongst the fragmented DNA. (C) Blebbing and nuclear margination (arrow marker) were noticed in 48-h treatment of Zm-Ag.NPs. Abbreviations**: **VC, viable cells; EA, early apoptotic cells; LA, late apoptotic cells

**Figure 11:**
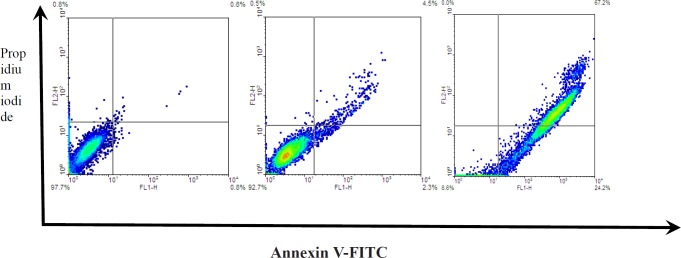
*Zm*-Ag.NPs induced apoptosis in HeLa cells. HeLa cells were treated with 10 and 20 μg/ml for 48 h. Phosphatidylserine (PS) externalization annexin V/propidium iodide (PI) double staining kit

**Figure 12:**
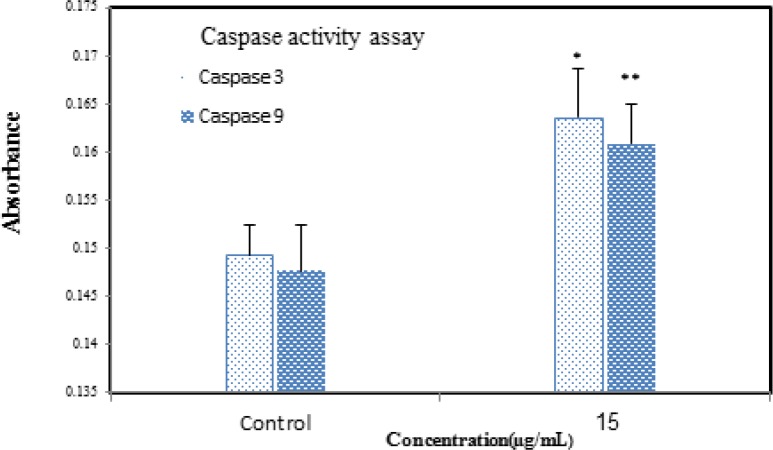
The activity of caspases -3/9 was increased after treatment, which indicates that apoptosis is significant (*p*>0.05). Data are presented as mean (± S.D.).

**Figure 13:**
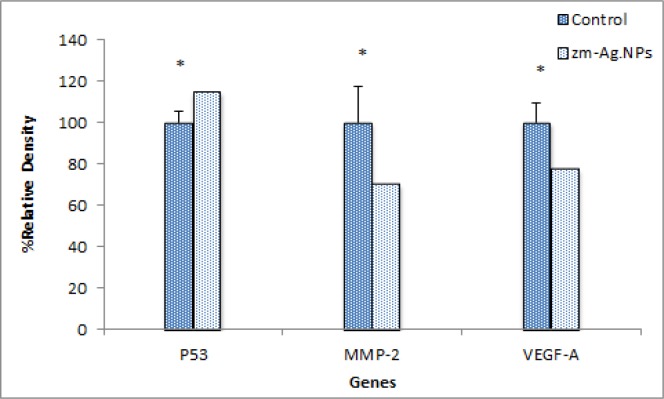
Scanning densitometry of semi-quantitative RT-PCR products for P53 gene matrix metalloproteinase MMP-9 and VEGF-A (*p *> 0.05). Data are presented as mean (±S.D

*EDS proﬁle of synthesized silver nanoparticles*

Energy dispersive spectroscopy (EDS Analysis) was used in conjunction with the scanning electron microscope (SEM), providing chemical analysis in areas as small as 1 µm in diameter. EDS detects all elements except H, He, Li and Be. EDS can be performed exactly on any feature or particle seen in the SEM images and can “MAP” elements on a surface. Unknown materials can be identified and quantitative analysis can be performed. The synthesis of silver nanoparticles using the extract of *Zm *leaves was characterized by EDS analysis, which provides evidence for the reduction of silver nitrate to elemental silver. The spectrum showed a strong silver signal together with about 3 kv with no contamination (Figure 3).

The FTIR measurement was done to identify the probable biomolecules responsible for the efﬁcient stabilization and capping of *Zm-*Ag.NPs synthesized using the extract of *Zm *leaves. The FTIR spectra for *Zm-*Ag.NPs are shown in Figure 4.

The spectrum in Figure 4 clearly indicates the adhesion of a residual *Zm *extract in the *Zm*-Ag.NPs as a capping agent. The *Zm-*Ag.NPs and plant extract FTIR spectra suggest the presence of various plant compounds, such as flavonoids and polyphenols, apart from other phytochemicals that were responsible for the formation of the *Zm-*Ag.NPs by reducing the Ag ions. The FTIR spectroscopic analysis confirms that the proteins present in the *Zm* leaf extract might act as a reducing agent, stabilize the *Zm*-Ag.NPs, and prevent agglomeration. Similar methods using the FTIR have been previously used to study *Gnidia glauca* flower extracts after bio reduction (16). The carbonyl group of amino acid residue has a strong binding affinity with metals; thereby, signifying the formation of a coated layer of *Zm-*Ag.NPs, which acts as a stabilizing agent to stop agglomeration in the aqueous medium.

*Transmission electron microscopy*

TEM is a powerful method to show the shape and size of the nanoparticles (17). The size and structure of the *Zm-*Ag.NPs are shown in Figure 5. The TEM image of *Zm*-Ag.NPs exhibits spherical (arrow marked 1), pentagonal (arrow marked 2) and undefined (arrow marked 3) shapes.

*Dynamic light scattering (DLS)* *study*

DLS is a technique that is often referred to as photon correlation spectroscopy, and is a common technique for determining the nanoparticle size in suspensions (18). The application of DLS in determining the size distribution of colloidal NPs in the range of 1 to 100 nm has been discussed previously. As shown in Figure 6, the DLS results showed that the distribution of *Zm*-Ag.NPs ranged from approximately 16 to 70 nm with an average size of 30 nm. This confirms that the sample contains various sizes of nanoparticle (Figure 6).

*Zeta potential measurement*

The zeta potential of a sample is most often used as an indicator of dispersion stability. A large zeta potential predicts a more stable dispersion. A high absolute zeta potential value indicates a high electric charge on the surface of the nanoparticles and the strong repellent forces amongst the particles, which prevent aggregation and lead to the stabilization of nanoparticles in the buffer solution. In natural conditions (pH close to 7.2), the values of zeta potential were equal to -10 to -40 mV. It could be concluded that *Zm*-Ag.NPs have a negative zeta potential and the particles are stable due to their electrostatic repulsion (Figure 7).

*Cell culture*

*Cytotoxity assay*

The growth inhibiting or inducing effects of various substrates on cell lines can be determined by the MTT assay. MTT is a membrane permeable dye that is metabolized to dark-blue crystals of formazan by mitochondrial dehydrogenases of living cells. After solubilization of the formazan crystals, the optical density (OD) of the dye was quantified using a multiwell-spectrophotometer at 570 nm. *Zm*-Ag.NPs were found to have the highest toxicity against HeLa cancer cells (IC_50_ = 15 μg/mL). Toxicity increases over time and in a concentration dependent manner, as the IC50 values of *Zm*-Ag.NPs were calculated as 15 μg/mL for 24 h and 10 μg/mL for 48 h respectively (Figure 8). Smaller *Zm*-Ag.NPs (5 to 30 nm) had a stronger cytotoxic effect, as they produced greater amounts of hydrogen peroxide (19). The average size of *Zm*-Ag.NPs used in this article was 30. Hence, this nanoparticle could penetrate the cell and lead to cell death efficiently (20).

*Apoptosis morphological identification with DAPI nuclear stain*

DAPI dye was applied to observe any nuclear change during apoptosis. When DAPI is bound to the DNA, it increases its fluorescence intensity approximately 20 fold higher than that of unbound DAPI. As the apoptotic cell membrane is compromised, more DAPI enters the cell and produces a stronger blue color stain. The differing nuclear morphology of apoptotic cells, such as chromosome condensation and fragmentation, also helps in the visual identification of the apoptotic cells stained with DAPI (23). The results showed that *Zm*-Ag.NPs lead to a breakdown in chromatin, as shown in Figure 9. Previously, the cytotoxicity biosynthesis of Ag.NPs from *S. cumini* extract on Dalton lymphoma cell lines were investigated and it was revealed that Ag.NPs can generate ROS that breaks down DNA, a hallmark in cells that appears to be undergoing apoptosis induction (Figure 9).

*Acridine orange (AO) /propodiumiudid (PI)*

Acridine orange (AO) is a metachromatic dye through which single-stranded (ss) stains emit green fluorescence in the interaction with double-stranded (ds) DNA, whereas for intercalation with DNA extraction, it is red fluorescence. Chromatin break down is an event of apoptosis that could be indicated with AO staining. Propodiumiodide enters into the cells that have lost their membrane integrity and indicates necrotic or late apoptosis cells (Figure 10).

*AnnexinV/PI*

In normal conditions, phosphatidylserine (PS) residues are found in the inner membrane of the cytoplasmic membrane in cells. During apoptosis, the PS becomes externalized. Annexin-V specifically binds to the PS, and, hence, the protein can be applied to detect apoptotic cells. Annexin-V conjugated to a fluorochrome dye is available. In this study, the effect of *Zm-*Ag.NPs on the apoptosis of HeLa cells was evaluated using the annexin V/PI double staining method. The results indicated that, following the treatment of HeLa cells for 48 h with 10 and 20 µh/mL Ag-NPs, the number of early apoptotic cells increased to 2.3 and 24.2, respectively, in 48 h. The cells in the late apoptosis stage increased to 4.5 and 67.2. The results revealed that *Zm-*Ag.NPs induced apoptosis in HeLa cells. Other investigations also proved that the apoptotic indexes indicated that *Zm*-Ag.NPs exhibited an apoptotic effect in a concentration-dependent manner (21) (Figure 11).

*Caspase activity*

Caspases are families that are important regulators of apoptosis. Caspases cleave to various substrates that lead to the typical biochemical and morphological changes in apoptotic cells, including cell shrinkage, chromatin condensation, DNA fragmentation, and plasma membrane blebbing. Thus, detection of caspase activation is important to determine the biological processes (22). Caspase-dependent apoptosis is the well-described modality of programmed cell death. Caspase activation in cells treated with *Zm-*Ag.NPs for 24 h was assessed. Caspase-3 and caspase-9 were activated by *Zm*-Ag.NPs. Gurunathan *et al*. (23) evaluated the potential toxicity of biosynthesized *Zm*-Ag.NPs in MDA-MB-231 human breast cancer cells. They indicated an increased level of caspase 3 activation in the treated cells. This result is in conformity with that of the current study (Figure 12).

*Gene expression by RT-PCR.*

*Gene expression analysis*

Matrix metalloproteinases (MMPs) are associated with cancer-cell invasion and metastasis. Tumor cells utilize the matrix degrading capability of these enzymes to spread to distant sites. They also act as regulatory molecules like adhesion molecules that modulate the bioavailability growth factors, such as VEGF to generate fragments with enhanced or reduced biological effects. In addition, MMPs are also thought to promote the growth of these tumor cells by activating cell metastasis. Of the various MMPs thought to be involved in cancer, attention has focused on the gelatinases, because (i) they are overexpressed in a variety of malignant tumors and (ii) their expression and activity are often associated with tumor aggressiveness and a poor prognosis (23). In this study, the inhibitory effects Zm-Ag.NPs on down regulation of MMP-2 and VEGF expressions were examined. The results of electrophoresis in agarose gel revealed a significant reduction (*P *˂ 0.05) in the MMP-2 gene expression (mRNA level). The level of P-53 after treatment increased dramatically but was not statistically significant (Figure 13). 

## Conclusion

One-step green synthesis of *Zm*-Ag.NPs is presented using the biodegradable *Z. multiflora* leaf extract. The diameter of the* Zm*-Ag.NPs was in the range of 16 to 70 nm, as shown by the DLS. The flavonoid, terpenoid, and protein constituents, which were present in the *Zm* leaf extract, were the surface active molecules stabilizing the nanoparticles. The hydroxyl and carboxylate groups of the extract facilitate reduced elemental silver. This proposed mechanism is also substantiated by the FTIR data. In this study, the toxicity effect of the biosynthesis of *Zm*-Ag.NPs as a potential agent for chemotherapy on HeLa cancer cells was assessed. The results indicated that treatment of HeLa cells with *Zm*-Ag.NPs leads to an increase in the expression of P53 protein and a decrease in the expression of VEGF and MMP-2. These proteins are involved in the progress of cancer. *Zm*-Ag.NPs also increases the activity of procaspase-9 and caspase-3. The involvement of Zm-Ag.NPs in induction of apoptosis leads to an increase in caspase-3/9 activity. Its effect on apoptosis was further confirmed by measuring its activity and the induction of apoptosis was measured by flow cytometry. As DNA damage is a feature of apoptotic cell death, DNA breakdown was further confirmed using the DAPI staining. This Ag.NPs is a carrier of plant material such as phenolic compound that can act as anticancer agents.
